# Could Sex/Gender Differences in ACE2 Expression in the Lungs Contribute to the Large Gender Disparity in the Morbidity and Mortality of Patients Infected With the SARS-CoV-2 Virus?

**DOI:** 10.3389/fcimb.2020.00327

**Published:** 2020-06-09

**Authors:** Gregor Majdic

**Affiliations:** ^1^Veterinary Faculty, Institute for Preclinical Sciences, University of Ljubljana, Ljubljana, Slovenia; ^2^Institute of Physiology, Medical School University of Maribor, Maribor, Slovenia

**Keywords:** lung, ACE2, COVID 19, sex difference, androgens

## Abstract

COVID-19 morbidity and mortality have significant gender disparities, with higher prevalence and mortality in men. SARS-CoV-2 enters the lungs through the ACE2 enzyme, a member of the renin-angiotensin system (RAS). Although there are no data for the lung, the expressions of RAS components in other tissues are modulated by sex hormones, androgens, and estrogens. However, there are no data on sex-specific differences in ACE2 expression. If there is a sex difference in the expression of ACE2 in the lung, this could theoretically explain the gender disparity in COVID-19 disease. More importantly, although modulation of ACE2 will certainly not provide a cure for the COVID-19 disease, modulation of ACE2 by sex hormone modulators, if they affect the expression of ACE2, could potentially be developed into a supportive therapy for COVID-19 patients.

## Main Text

A novel coronaviral disease, COVID-19, caused by the SARS-CoV-2 virus (Guo et al., 2020), is affecting a disproportionally higher number of men than women. Epidemiological data show that a much larger number of men are severely affected by the disease, and there is an even more substantial gender difference in the mortality of patients with COVID-19. This large gender difference has been shown both in China (Guan et al., 2020; Jin et al., 2020), the first country affected by COVID-19, and recently in Italy (Riccardo et al., 2020), a country that is currently, in the middle of March 2020, the most affected by this disease. A preprint reporting a meta-analysis of 39 reports, including 206,128 patients, confirms the sex bias, with much higher mortality and more severe presentation of the disease in men in several countries affected by COVID-19 throughout the world (Peckham et al., 2020). Age is another risk factor for both morbidity and mortality in COVID-19 patients, with children seemingly mostly resistant to the effects of the SARS-CoV-2 virus (Lu et al., 2020). Various hypotheses for explaining sex differences in morbidity and mortality due to COVID-19 disease have been proposed, from the biological, such as innate differences in the male and female immune system, to the environmental, such as a larger number of males smoking (Cai G. 2020). These differences are also sometimes proposed as explanations for the relative resistance to the disease by children, as children do not smoke, and their immune system is markedly different from the adult immune system.

However, none of these hypotheses have been proven so far. Although differences in the immune system might account for the differences in the morbidity and mortality between men and women, there are so far no studies to describe or propose how the male and female immune systems might interact differently with SARS-CoV-2. The smoking hypothesis has been disputed, as a relatively low number of patients were smokers, even in China, where smoking is much more prevalent than in western countries (Cai H. 2020). Furthermore, in Italy, previous epidemiological studies did not report significant gender differences in smoking (Sardu et al., 2009) that could contribute to the substantial gender differences in susceptibility to SARS-CoV-2.

Here, I propose a novel hypothesis that not only addresses the significant gender differences in morbidity and mortality due to COVID-19 but also potentially tackles the low morbidity and especially the low mortality in children infected by the SARS-CoV-2 virus.

As shown by several studies, the most likely entry point for SARS-CoV-2 is angiotensin-converting enzyme 2 (ACE2) (Guo et al., 2020; Tai et al., 2020; Zhang et al., 2020). The SARS-CoV-2 virus has a spike protein that interacts with ACE2; this is similar to the SARS-CoV virus, which enters the cells through ACE2. Notably, the spike protein is very similar in SARS-CoV-2 and SARS-CoV, strongly supporting the role of ACE2 as an entry point for the virus (Tai et al., 2020; Zhang et al., 2020).

ACE2 enzyme is part of the renin-angiotensin pathway that plays important roles in the regulation of fluid homeostasis in the body and is present in various epithelial cells, including lung and respiratory tract (Kuba et al., 2006). ACE2 homolog angiotensin-converting enzyme (ACE) plays a crucial role in this system by cleaving angiotensin I into angiotensin II. Angiotensin II is a small peptide with strong effects on vasoconstriction and sodium balance. ACE2 cleaves angiotensin I and II into smaller peptides that seem to cause vasodilatation and thus counteract the action of angiotensin II.

Cardiovascular diseases are much more prevalent in men than in women (Ventura-Clapier et al., 2017). The main underlying cause of this seems to be female exposure to estrogens, as risk of cardiovascular diseases increases in women after menopause. It is not yet known through which mechanisms estrogens exert protective effects on cardiovascular health. Several previous studies have shown that the sex hormones androgens and estrogens influence the renin-angiotensin system (Reckelhoff, 2001; McGuire et al., 2007; Rabi et al., 2008; White et al., 2019). Androgens increase plasma renin activity and expression of angiotensinogen messenger RNA (Reckelhoff, 2001), while estrogens decrease plasma renin activity, decrease angiotensin I receptor expression, and decrease the expression of angiotensin-converting enzyme 1 (McGuire et al., 2007). One study also reported higher activity (but not expression) of ACE2 in male mouse kidneys and adipose tissue in comparison to female mice (Gupte et al., 2012). Recently, an *ad hoc* study on previously collected datasets did not find sex differences in ACE2 mRNA expression between males and females (Cai G. 2020), but this was performed on samples collected for a different study and cannot be viewed as definitive proof, especially as the study did not examine either the protein expression or activity of ACE2 enzyme.

Therefore, I propose the hypothesis that the expression of ACE2 protein is different between males and females and that this sex difference contributes to the gender disparity in morbidity and mortality from the COVID-19 disease. I also propose that sex hormones modulate sex differences in the expression of ACE2 in lung and that modulating the expression of ACE2 in lung by sex hormone modulators (anti-androgens, anti-estrogens) could influence the COVID-19 disease.

To test this hypothesis, the following studies should be performed:

1. Examine in prospective planned studies whether there are sex differences in the expression of gene and protein ACE2 in lung in both human lung samples and laboratory animals, preferably cats or ferrets, as these animals are the most suspectable to the SARS-CoV-2 virus (Shi et al., 2020).
2. Examine whether ACE2 expression in lung is regulated *in vivo* by the sex hormones testosterone and estradiol in animal models.
3. If either testosterone or estradiol regulates ACE2 expression, examine whether modulators of these hormones such as testosterone or estradiol antagonists affect the expression of ACE2 in lung cells in animal models.
4. From epidemiological data available, examine whether there is any effect of using antiandrogens (such as in patients with prostate cancer) or estrogens (postmenopausal women using hormone replacement therapy).

If the results of the proposed studies suggest sex differences in and sex hormone modulation of the ACE2 enzyme, this could pave the way to utilizing these findings in clinical patients. Clearly, just modulating the expression of ACE2 in the lungs will not prevent a person from contracting the disease or cure COVID-19, but it might help to alleviate the viral load and severe symptoms in male patients. Furthermore, if sex hormones indeed modulate the expression of ACE2 in the lungs and thus contribute to the development of COVID-19 disease, this could explain low morbidity in children, especially in prepubertal children, in whom levels of sex hormones are very low.

## Data Availability Statement

The original contributions presented in the study are included in the article/supplementary materials, further inquiries can be directed to the corresponding author/s.

## Author Contributions

GM prepared and wrote the entire manuscript.

## Conflict of Interest

The author declares that the research was conducted in the absence of any commercial or financial relationships that could be construed as a potential conflict of interest.
